# Consumers' Kansei Needs Clustering Method for Product Emotional Design Based on Numerical Design Structure Matrix and Genetic Algorithms

**DOI:** 10.1155/2016/5083213

**Published:** 2016-08-18

**Authors:** Yan-pu Yang, Deng-kai Chen, Rong Gu, Yu-feng Gu, Sui-huai Yu

**Affiliations:** ^1^School of Construction Machinery, Chang'an University, Xi'an 710064, China; ^2^Department of Industrial Design, Northwestern Polytechnical University, Xi'an 710072, China

## Abstract

Consumers' Kansei needs reflect their perception about a product and always consist of a large number of adjectives. Reducing the dimension complexity of these needs to extract primary words not only enables the target product to be explicitly positioned, but also provides a convenient design basis for designers engaging in design work. Accordingly, this study employs a numerical design structure matrix (NDSM) by parameterizing a conventional DSM and integrating genetic algorithms to find optimum Kansei clusters. A four-point scale method is applied to assign link weights of every two Kansei adjectives as values of cells when constructing an NDSM. Genetic algorithms are used to cluster the Kansei NDSM and find optimum clusters. Furthermore, the process of the proposed method is presented. The details of the proposed approach are illustrated using an example of electronic scooter for Kansei needs clustering. The case study reveals that the proposed method is promising for clustering Kansei needs adjectives in product emotional design.

## 1. Introduction

Companies that listen to their consumers are more likely to be successful in the market [1, 2]. Understanding and interpreting consumers' needs have become an indispensable part in new product development (NPD) process [3]. With the transformation from industrial economy to experience economy, companies focusing solely on functionality and usability may not win prominent success because consumers' needs gradually changed from traditional functional needs to emotional needs nowadays [4–8]. As a result, consumers buy products not only for basic function and usability, but also for emotional experiences that could be evoked by attractive product appearance [9]. As Gobe [10] pointed out today's market competition is dominated by emotional sense, and the brand's most important investment is to create the right emotional atmosphere. When coming to product design, involving emotional elements in product design could make a product attractive and evoke positive emotions [11]. In this regard, Kansei engineering [4, 5] has been developed as a comprehensive psychological and ergonomic consumer-oriented technique to help designers to link consumers' emotional response to design properties of a product [12]. Kansei engineering consists of five types [4, 13]: (1) category classification for identifying consumer's affective need with a tree structure; (2) Kansei engineering system (KES), a computer aided system with mathematically statistical tools to connect Kansei and product properties; (3) hybrid engineering system, incorporating KES and Kansei prediction elicited by product properties; (4) Kansei engineering modeling for assessing consumers' feeling of Kansei words; (5) virtual Kansei engineering with a virtual reality system for presenting products and standard data collection systems. The processing of Kansei needs may vary depending on different types, but they all focus on translating consumers' Kansei needs into product properties. Kansei engineering has been successfully applied in various fields of product design, such as alarm clock [14], sunglasses [15], digital camera [16], mobile phone [17], and domestic commodities [18].

Consumers' Kansei needs refer to the senses of sight, hearing, feeling, smell, taste, recognition, and balance that a product evoked [8], and these needs can be measured by physiological response, behaviors and actions, and psychological methods. A specific Kansei arises when a consumer is subjected to a product and is always described with perceptual words or so-called Kansei adjectives, such as beautiful, fashionable, and complicated. These words reflect consumers' individual feeling and subjective impression about a product and have been most commonly used as a psychological method to describe the product domain when measuring Kansei needs. Consumers' Kansei needs are affected by multiple factors, including cultural background, personal experience, and lifestyle, and they can be changed in different contexts. Thus, to cover all the possibilities, all available sources should be involved to collect Kansei adjectives, including literatures, manuals, experts, experienced users, ideas, and visions.

A good result should include all important words and the number varies from 50 to 600 words [6]. However, it is very difficult for industrial designers to deal with all the Kansei needs. The key issue is reducing the dimension of the complex emotional needs to fewer Kansei adjectives through data analysis and clustering, providing a more accurate basis for industrial designers. In this regard, clustering algorithms are exploited to solve this problem. Conventional clustering methods can be mainly categorized into hierarchical clustering and partitional clustering [19]. Hierarchical clustering refers to building a hierarchy of clusters by “bottom up” approach as agglomerative hierarchical clustering and “top down” approach as divisive hierarchical clustering [20]. Partitional clustering is defined as partitioning data into several parts, each part as a cluster with prespecified function. With the advantages of hierarchical clustering methods, Choi and Jun [21] implemented hierarchical clustering of Kansei factors of tactile sense by studying product surface roughness and proposed several criterion functions to generate optimal clustering number of Kansei adjectives. On the other hand, Zhou et al. [22] constructed a partitional clustering algorithm, *k*-means, to obtain the quantitative relationship between the product parameters and the Kansei characteristics of similar consumers. Additional clustering methods were also utilized to dispose Kansei needs. For instance, Factor analysis and Procrustes analysis were combined to extract representative Kansei adjectives [16], and a design structure matrix (DSM) was used for Kansei needs clustering by differentiating them for a serial of subsets so that they could be taken separately and easily by participants [23].

However, hierarchical clustering methods may ignore relatively weak connections among Kansei needs and partitional clustering methods require Kansei adjectives mapping in a continuous region, which make them not suitable for clustering Kansei needs [23]. Fuzzy *C*-Means (FCM) algorithm [24], the most widely used fuzzy clustering method, can be capable of processing vague data such as Kansei needs by grouping data elements into more than one cluster. Nonetheless, the clustering result depends largely on the initial weights and appropriate membership function and thus leads to low clustering reliability. A combined DSM [23] was promising in clustering Kansei needs, but the clustering efficiency and reliability may be affected by various consumers' viewpoints and it is not efficiency to find out the optimum clusters.

While solving the above problems and establishing relationships among Kansei needs, this study contributes to improving the efficiency of Kansei needs clustering and finding optimum clusters by combining numerical design structure matrix (NDSM) and genetic algorithms. It makes use of a conventional DSM structure to address and quantify the relationship among Kansei needs with a four-point scale method. Essentially, genetic algorithms are applied to encode Kansei needs and implement clustering automatically, aiming to find optimum clusters through decoding the optimum chromosome. The proposed method incorporates three parts: (1) choosing appropriate Kansei words that could describe target product from all available sources, (2) establishing an NDSM by dissecting and representing connection intensity of every two adjectives, and (3) using genetic algorithms to find optimum clusters of the Kansei needs NDSM.

## 2. Methods

### 2.1. Introduction to NDSM

**Figure 1:**
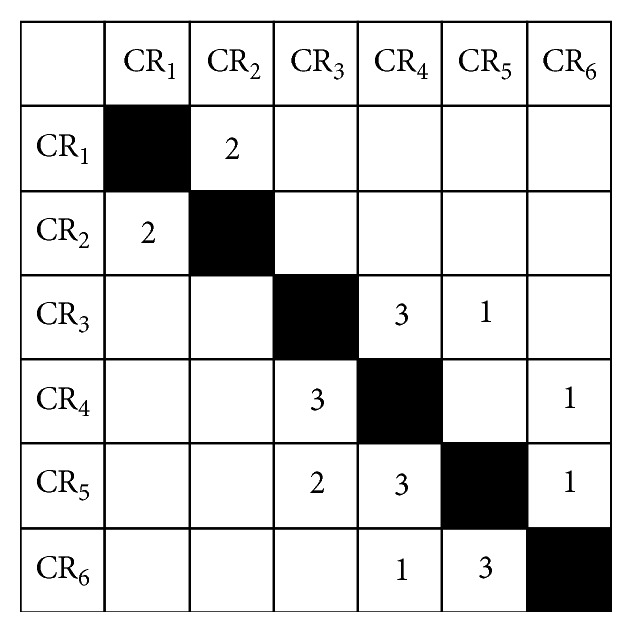
A typical NDSM.

The DSM is a square matrix tool developed by Dr. Steward and can be applied to product development analysis and planning [25]. It is developed from directed graph initially and has the strength of representing a large number of constituent elements and the corresponding information exchange. In a DSM, the matrix elements represent activities in product development, while the off-diagonal cells are used to indicate relationships between every two units, in which the cells below the diagonal stand for feed-forward relationship and above the diagonal indicate feedback relationship. The cells along the diagonal are senseless. Data in cells are represented in a binary way, such as “0”, “1”, “×”, and “NULL”. On this basis, Eppinger [26] and Smith and Eppinger [27] put forward numerical design structure matrix (NDSM) to quantitatively describe the strength of the relationship between the matrix elements. A typical NDSM is shown in Figure 1.

As shown in Figure 1, off-diagonal values indicate weights of two related elements by four-point scale method. Weights greater than or equal to 2 represent a strong link between two elements, while weights greater than 0 and less than 2 mean weak relationships, and weights equal to 0 (blank units) indicate no connection.  Table 1 shows weights and their meanings of four-point scale method.

Clustering an NDSM can be achieved by steps of changing the sequence of units. For a Kansei needs set CR = {CR_1_, CR_2_,…, CR_*m*_},  CR_*ij*_  (*i*, *j* = 1, 2, …, *m*) is a cell value in row *i* and column *j* in the Kansei needs NDSM, representing the link weight between CR_*i*_ and CR_*j*_. Supposing that *M* is a 1 × *m* row vector and *M*′ is *m* × 1 column vector, matrix transformation of the NDSM can be performed as follows.

*(1) Change the Sequence of Row Elements*. Consider

(1) M=CRi1,CRi2,…,CRimT,CRi1,CRi2,…,CRimT=CRj1,CRj2,…,CRjmT,CRj1,CRj2,…,CRjmT=M.

*(2) Change the Sequence of Column Elements*. Consider

(2) M′=CR1i,CR2i,…,CRmiT,CR1i,CR2i,…,CRmiT=CR1j,CR2j,…,CRmjT,CR1j,CR2j,…,CRmjT=M.

Clustering process depicts the following steps.

**Step 1 .**

Identify independent elements that have no link with any other elements and place them in front of all row and column elements. They do not need clustering.

**Step 2 .**

Identify Bus elements that have links with most other elements and place them in the rearmost part of the NDSM. They do not need clustering, either.

**Step 3 .**

In addition to independent elements and Bus elements, choose any element and find out other elements that have strong and less strong link with it, and then perform the same operation to these elements until there are no remaining elements suitable for this purpose. Take these elements as one cluster.

**Step 4 .**

Repeat Step 3 to cluster the remaining elements till all elements have been placed into clusters.

**Step 5 .**

Add weak links to the clustered matrix.

### 2.2. Genetic Algorithms for Clustering an NDSM

As shown above in Section 2.1, the key clustering step is repetitively executing sequence transformation of the NDSM elements, which will involve substantial expenditure of time and energy for industrial designers to seek out optimum clusters. Thereupon, it is essential to choose appropriate optimization method for aiding clustering. The mainstream optimization methods include neural network (NN), support vector machine (SVM), and genetic algorithms (GAs), and all of them have been applied to product design process successfully, such as NN for predicting the best product form combination [28], SVM for establishing and dealing with the precise relationship between product form elements and images (customers' affective feelings) [3, 17], and GAs for assessing product design feasibility to derive optimum solutions [18]. Among them GAs perform search according to a specified fitness function and the experience of previous generation and are more effective at searching the entire solution space comparing to NN and SVM [18, 29]. Therefore, the GAs are applied in this study.

*(1) Construct the Initial Population*. To cluster a Kansei needs set CR = {CR_1_, CR_2_,…, CR_*m*_}, linguists and experienced industrial designers are organized to work together to rate every two Kansei adjectives on a scale of 0 to 3 as shown in Table 1. A Kansei needs NDSM then can be established with means of scores filling in each unit. Set *k* as the maximum number of clusters. Then a two-dimensional matrix is coded as follows:

(3) $$\begin{array}{c} CL=\begin{array}{cc} \begin{array}{c} CL_{1}\\ CL_{2}\\ \vdots \\ CL_{k} \end{array} & \begin{array}{c} \begin{array}{cccc} CR_{1} & CR_{2} & \cdots & CR_{m} \end{array}\\ \left\{\begin{array}{cccc} c_{11} & c_{12} & \cdots & c_{1m}\\ c_{21} & c_{22} & \cdots & c_{2m}\\ \vdots & \vdots & \ddots & \vdots \\ c_{k1} & c_{k2} & \cdots & c_{km} \end{array}\right\} \end{array} \end{array}, \end{array}$$

where each row represents a cluster and each column represents one element corresponding to the NDSM. *c*_*ij*_  (*i* = 1, 2, …, *k*, *j* = 1, 2, …, *m*) means whether CR_*j*_ belongs to cluster CL_*i*_ or not, and its value is obtained by

(4) $$\begin{array}{c} c_{ij}=\left\{\begin{array}{cc} 1, & CR_{j}\in CL_{i}\\ 0, & CR_{j}\notin CL_{i}. \end{array}\right. \end{array}$$

Population initialization steps are as follows.

**Step 1 .**

For a chromosome CL, take an element of each column vector and assign the value to 1 and the remaining value of 0.

**Step 2 .**

Repeat Step 1 until accruing sufficient initial chromosomes. Population size is determined by multiples of number of row or column elements in Kansei NDSM empirically.

*(2) Define the Fitness Function*. Three prerequisites should follow when defining the fitness function: (1) the link between intracluster elements should be as large as possible whereas the link between clusters should be as little as possible; (2) links in a small cluster contact easier than a larger one; (3) along with the number of clusters increasing, the difficulty of managing clusters increases accordingly.

In the population of any generation, chromosome *h* consists of *k* clusters, and weight of gene *j* in cluster *i* in relation to other genes with value of 1 can be calculated by

(5) $$\begin{array}{c} w_{ij}=\left(\;c_{ij}\cdot c_{ig}\;\right)\cdot \overset{m}{\underset{g=1}{\sum }}CR_{jg}, \end{array}$$

where *c*_*ij*_ and *c*_*ig*_ represent weight of genes *j* and *g* in cluster *i* of chromosome *h* and CR_*jg*_ is the link weight of elements in the Kansei NDSM corresponding to *c*_*ij*_ and *c*_*ig*_.

If cluster *i* consists of *m*_*i*_ elements (all elements have the value 1), CL_*i*_ can be computed by

(6) $$\begin{array}{c} CL_{i}=\frac{1}{m_{i}}\overset{m_{i}}{\underset{j=1}{\sum }}w_{ij}. \end{array}$$

If chromosome *h* consists of *k*_*k*_ clusters actually, then its holistic cluster value is

(7) $$\begin{array}{c} CL\left(\;h\;\right)=\overset{k_{k}}{\underset{i=1}{\sum }}CL_{i}. \end{array}$$

Taking CL(*h*) as the objective function, fitness function is proposed as

(8) $$\begin{array}{c} f\left(\;h\;\right)=\frac{CL\left(h\right)-CL_{min}}{CL_{max}-CL_{min}}, \end{array}$$

where CL_min_ and CL_max_ represent min and max value of objective function, respectively.

In accordance with the method of roulette, selected probability of chromosome *h* is calculated by

(9) $$\begin{array}{c} p\left(\;h\;\right)=\frac{f\left(h\right)}{\sum_{x=1}^{y}f\left(x\right)}, \end{array}$$

where *f*(*x*) is the fitness value of chromosome *x* and *y* is total number of all chromosomes.

*(3) Crossover and Mutation*

*Crossover.* Perform one-point crossover by selecting two parent chromosomes randomly and setting the same column as crossing point. When implementing crossover, exchange the right parts of crossing points in two parent chromosomes and thus generate two new individuals.

*Mutation*. Select any two columns of a parent chromosome to exchange.

**Figure 2:**
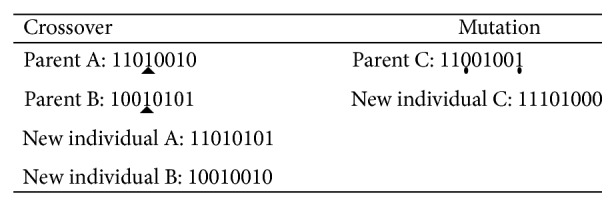
An example of crossover and mutation operation.

An example of crossover and mutation operation is shown in Figure 2.

### 2.3. Kansei Needs Clustering Process

Based on NDSM and genetic algorithms, Kansei needs clustering process can be presented as follows. 

*(1) Collecting Specimens of Target Product and Corresponding Kansei Adjectives*. Specimens of target product and likewise product are collected from websites, and high similarity samples are removed through preliminary analysis. Meanwhile, Kansei adjectives are collected from all available sources where they are used to describe the target product domain. Sources may include websites, literatures, product manuals, magazines, experts, industrial designers, experienced users, and dissertations. Kansei adjectives should include important words that can be used to describe product samples as many as possible. Adjectives and their antonyms are paired up if they both occur, or find the right antonym for each Kansei adjective to form a pair.

*(2) Investigating Consumers*. A web-based questionnaire is designed integrating target product domain and Kansei adjectives. Likert scale method is used to survey consumers' preferences of adjectives with respect to each target product.

*(3) Analyzing and Filtering Kansei Needs*. The data collected by valid questionnaires is statistically and correlatively analyzed, in which irrelevant words to consumers' preferences are removed and complete correlative adjectives should be amalgamated into single one. The filtering Kansei adjectives are depicted as CR = {CR_1_, CR_2_,…, CR_*m*_}.

*(4) Constructing Kansei NDSM*. According to four-point scale method shown in Table 1, linguists and experienced industrial designers work together to rate every two Kansei adjectives on a scale of 0 to 3. An NDSM then can be established with mean of scores.

*(5) Clustering Kansei Needs Based on NDSM and Genetic Algorithms*. The Kansei NDSM can be clustered by changing sequences of row and column elements as well as with (3) to (9).

*(6) Extracting Primary Needs*. According to the Kansei NDSM after clustering, linking diagrams of each cluster can be charted to extract predominant factor of each cluster, aiming to provide more accurate design basis for industrial designers.

## 3. A Case Study

**Figure 3:**
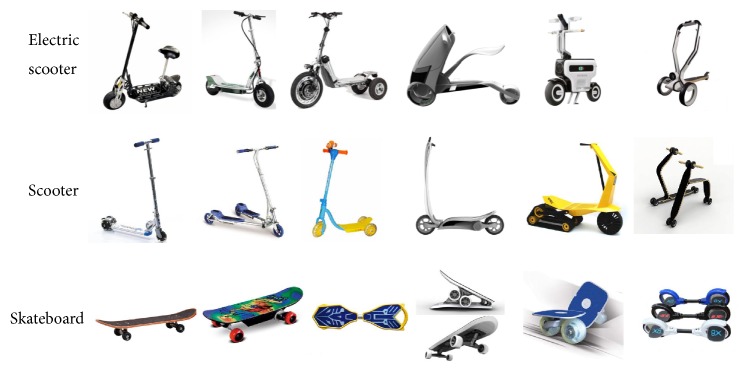
Product samples.

An experiment of clustering user's Kansei needs about electric scooter was conducted to illustrate how the proposed method worked. The eight juniors at Chang'an University majored in industrial design joined to the market research and 93 middle school students and 116 high school students (boys 68.3% and girls 31.7%, ages between 13 and 19) evaluated the samples using questionnaires. To collect users' Kansei needs effectively, electric scooters as well as similar products were involved in this research, including scooters and skateboards, covering current production and concept design (seen in Figure 3). The fifty-five Kansei adjectives about product samples were collected from websites, literatures, product manuals, magazines, experts, industrial designers, experienced users, and dissertations. Adjectives with antonyms were paired up and others were endowed with right antonyms, and based on this a seven-point Likert scale was used to evaluate customers' response about product samples. The response rate was 93.3% and 195 questions were used. Combined with user interviews and correlation analysis, 49 Kansei adjectives were obtained, shown as follows:

 simple, modern, outstanding, exquisite, manly, innovative, free, speedy, lively, plump, elaborate, futuristic, special, practical, abrupt, integral, mellow, bright, hard, glossy, vivid, organic, fluent, energetic, powerful, precise, classy, orderly, decent, beautiful, coordinating, harmonious, distinct, sportive, striking, bionic, avantgarde, fickle, dynamic, technological, likeable, saponaceous, gorgeous, heavy, abstract, overbearing, comic, agile, and tensional.

By communicating with three industrial designers, “practical” may reflect a demand in use and was seen as an independent element, which would not be involved in clustering. The three industrial designers and a linguist were asked to analyze and rate every two Kansei adjectives on a scale of 0 to 3 according to Table 1. A Kansei needs NDSM was built with each unit filling with a corresponding average score, shown in Table 2 (diagonal entries were assigned with 0).

**Figure 4:**
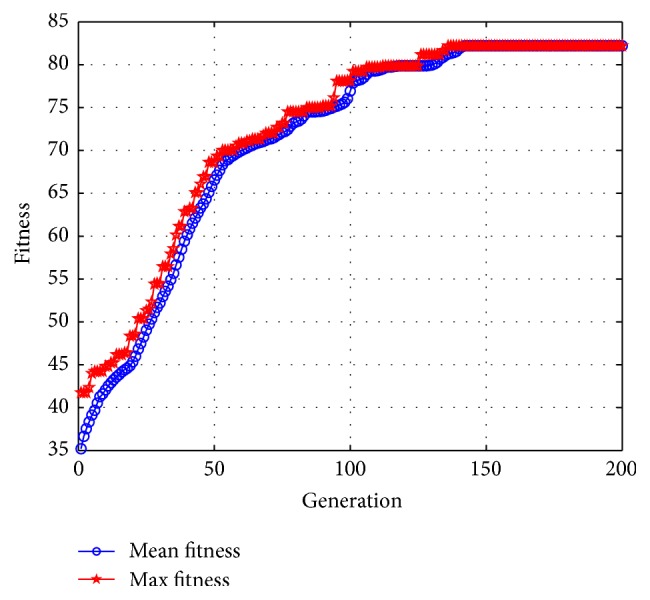
Converging curved lines.

The Kansei needs NDSM consists of 48 adjectives. Maximum number of clusters may affect computing efficiency and too many may be intractable for industrial designers to handle, while inadequate clusters may result in imperfect description of product images. Through repeated experiments with different number of clusters (computing results shown in Table 3), combined with opinions from 3 industrial designers who participated in constructing the Kansei NDSM, 6 clusters are suitable for depicting consumers' perceptual needs toward electric scooter and can be easily addressed. As a result, chromosome was coded into a 6 × 48 matrix with (3). Crossovers and mutations were set at 80% and 20%, respectively. Clustering algorithms undertook 200 generations with a population size of 96 using a MATLAB program. Converging curved lines reflected that computing converged at generation 142 with objective function value of 82.2049, shown in Figure 4. Through clustering, adjectives with weak connections have been isolated and similar adjectives with strong connections are placed together, resulting in six clusters, shown in Table 4.

**Figure 5:**
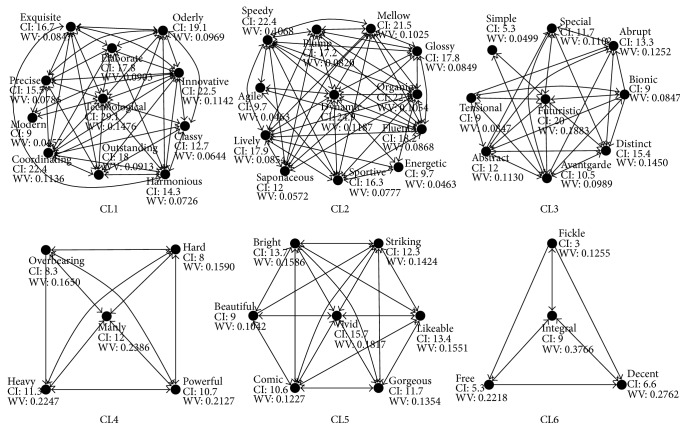
Node linking diagram.

According to internal link of each cluster, linking graph was depicted for identifying primary adjectives in each cluster, shown in Figure 5. Each node represents a Kansei adjective and each arrow means feed-forward or feedback relationship. An arrow going forth means an initiative interrelation by an adjective referring to another adjective, while going back means passive interrelation. Then connection intensity (CI) of each node can be calculated and the weight value (WV) for each Kansei word is computed using the following formula: WV_*i*_ = CI_*i*_/∑_*i*=1_^*n*^CI_*i*_, where *n* represents the number of elements in a cluster. A node in a cluster with maximum connection intensity of arrows going forth is defined as an initiative node, which has the largest impact on other nodes. As shown in Figure 5, these nodes are CL1/technological, CL2/dynamic, CL3/futuristic, CL4/manly, CL5/vivid, and CL6/integral. Locating in the center of each cluster, these nodes are key nodes and should be given high priority by designers as primary Kansei needs.

## 4. Discussion

As shown in Figure 5, each cluster comprises several Kansei adjectives with a center node. The following part explains the meanings among them.

1. In cluster CL1, “technological” has maximum feed-forward connection intensity with others, followed closely by “innovative” and “coordinating,” while “modern” has the least. This may imply more technological elements and innovative function to be incorporated in electric scooter design in a coordinating manner.
2. Cluster CL2 has the most Kansei adjectives with “dynamic” as a center element. The connection intensity of “dynamic” is 24.9 and weight value accounts for more than 11 percent. In addition, “speedy” and “organic” are after the center node, and they three make up almost a third of all CI in this cluster. They should be considered simultaneously for forming dynamic style.
3. “Futuristic” has no connection with “abrupt” in CL3, which means they may conflict in expressing Kansei needs. Meanwhile, “avantgarde” can lead to “abrupt” and in turn will not do so, the same thing with “tensional” to “abrupt.” The same happens between “abstract” and “distinct,” “abrupt” and “distinct,” “abstract” and “special,” “bionic” and “distinct,” and “bionic” and “abrupt.”
4. The five Kansei words alike in semantic in CL4 have uniform connection intensity on each other except for “heavy” not leading to “overbearing.” They reflect volume style of an electric scooter and the center word implies the main users are boys, as the market research shows.
5. The center node in CL5 reflects whether the product is attractive in appearance, and vivid style should be considered first.
6. “Integral,” as the center of cluster CL6, which has the least Kansei adjectives, may mean most part of an electric scooter should be covered through form design to create an integral and organic whole.

## 5. Conclusion

A novel method for consumers' Kansei needs clustering method based on numerical design structure matrix (NDSM) and genetic algorithms is proposed in this work. The method demonstrates the efficiency for reducing dimensions of Kansei adjectives by parameterizing a conventional DSM and integrating genetic algorithms to find optimum Kansei clusters. Therefore, a new NDSM is obtained in which Kansei adjectives are clustered along diagonal elements, which helps to clarify primary adjectives. The process of the proposed method is presented and illustrated using an example of electronic scooter for consumers' Kansei needs clustering. The results suggest that parameterizing a conventional DSM helps to quantify the correlation between every two Kansei adjectives. In addition, using genetic algorithms to cluster an NDSM and find optimum Kansei needs clusters is suitable. It appears that the proposed method is promising for clustering Kansei needs adjectives in product emotional design.

## Figures and Tables

**Table 1:** Weights and their meanings of four-point scale method.

Scale	Weights	Meanings
High	3	Strong link
Medium	2	Less strong link
Low	1	Weak link
Null	0	No link

**Table 2:** A Kansei needs NDSM.

		CR_1_	CR_2_	CR_3_	CR_4_	CR_5_	CR_6_	CR_7_	CR_8_	CR_9_	CR_10_	CR_11_	CR_12_	CR_13_	CR_14_	CR_15_	CR_16_
		Simple	Modern	Outstanding	Exquisite	Manly	Innovative	Free	Speedy	Lively	Plump	Elaborate	Futuristic	Special	Abrupt	Integral	Mellow

CR_1_	Simple	0.0	3.0	0.3	1.7	1.3	1.0	1.3	0.0	0.0	1.7	0.0	3.0	0.0	0.0	2.7	2.3
CR_2_	Modern	3.0	0.0	0.0	0.0	2.0	1.3	1.0	0.3	0.3	1.0	0.0	3.0	0.3	0.3	2.0	0.3
CR_3_	Outstanding	1.0	0.0	0.0	3.0	0.7	3.0	3.0	0.3	0.0	1.3	0.0	2.3	2.0	1.0	0.3	0.7
CR_4_	Exquisite	1.7	0.0	3.0	0.0	0.3	2.0	0.0	0.0	0.0	0.3	3.0	3.0	0.3	0.3	0.3	0.0
CR_5_	Manly	0.7	0.7	0.3	0.3	0.0	0.3	0.0	0.7	0.3	0.7	0.0	0.7	0.0	0.7	0.3	0.0
CR_6_	Innovative	1.0	3.0	3.0	1.7	0.0	0.0	2.0	0.3	0.7	0.0	2.3	2.3	1.3	0.0	1.3	0.3
CR_7_	Free	0.0	1.7	1.3	1.3	0.3	1.7	0.0	2.7	2.0	0.3	0.3	2.3	1.7	1.7	1.7	1.3
CR_8_	Speedy	0.0	1.7	0.7	0.0	2.0	0.3	2.0	0.0	2.3	0.3	0.3	2.3	0.3	0.3	1.3	2.7
CR_9_	Lively	0.3	1.7	0.0	0.3	1.7	0.0	2.0	2.3	0.0	2.0	0.7	1.3	0.3	0.0	1.3	2.3
CR_10_	Plump	0.3	0.3	1.7	0.3	1.0	0.3	1.3	0.3	1.7	0.0	0.3	0.0	0.0	0.3	1.3	3.0
CR_11_	Elaborate	2.3	0.0	0.0	3.0	0.3	3.0	0.7	0.0	0.7	0.0	0.0	0.7	0.7	0.3	0.3	0.7
CR_12_	Futuristic	3.0	3.0	2.0	0.3	2.0	2.3	2.7	3.0	0.3	1.3	1.3	0.0	1.0	0.0	0.0	0.0
CR_13_	Special	0.0	1.3	2.0	0.3	0.0	1.0	0.3	0.3	0.3	0.0	2.7	1.3	0.0	2.0	0.3	0.7
CR_14_	Abrupt	0.0	0.3	0.3	0.7	0.3	0.3	1.7	0.0	0.7	0.0	0.3	0.0	3.0	0.0	0.3	0.0
CR_15_	Integral	3.0	2.0	2.0	2.0	0.7	1.7	0.3	0.3	0.7	1.3	1.7	3.0	0.3	0.0	0.0	0.7
CR_16_	Mellow	2.3	1.0	1.3	0.3	0.3	0.7	0.3	0.3	1.3	2.7	1.0	3.0	0.0	0.7	1.3	0.0
CR_17_	Bright	0.7	1.3	2.7	1.7	0.0	0.3	0.3	0.7	2.3	0.3	1.7	0.3	0.3	0.3	0.3	0.0
CR_18_	Hard	0.7	0.3	1.3	0.3	3.0	0.3	0.7	0.3	0.3	0.7	0.3	0.3	0.3	1.3	0.7	0.0
CR_19_	Glossy	2.0	2.3	1.3	0.0	0.0	0.7	0.7	2.0	0.0	2.0	0.7	3.0	0.3	0.0	2.3	3.0
CR_20_	Vivid	0.3	2.0	2.0	0.0	0.3	0.3	0.7	0.3	2.7	0.3	1.0	0.3	0.0	0.0	0.3	0.3
CR_21_	Organic	1.7	2.3	0.3	1.3	0.3	2.3	2.3	1.3	2.7	1.7	1.3	2.0	0.0	0.3	2.7	2.3
CR_22_	Fluent	2.0	1.7	0.3	2.0	0.3	0.7	1.3	1.0	2.7	1.7	0.0	1.3	0.7	0.7	2.3	2.3
CR_23_	Energetic	0.0	0.0	2.3	0.3	2.3	0.0	2.3	0.0	3.0	0.0	0.7	1.3	0.7	0.0	0.3	2.0
CR_24_	Powerful	0.7	0.0	0.7	0.3	3.0	0.7	0.7	0.0	0.3	0.3	0.0	0.3	0.7	1.7	0.7	0.7
CR_25_	Precise	0.3	2.7	2.7	3.0	2.7	2.3	0.3	0.7	0.0	0.3	2.7	3.0	0.0	0.7	0.7	0.0
CR_26_	Classy	3.0	3.0	3.0	0.0	2.7	3.0	0.7	1.0	0.0	0.3	0.0	3.0	0.0	0.3	1.0	1.3
CR_27_	Orderly	3.0	0.0	0.0	1.0	1.7	1.3	2.3	2.0	1.3	0.7	1.3	0.3	0.7	0.7	2.0	2.0
CR_28_	Decent	3.0	0.0	0.3	1.0	0.3	0.3	0.0	0.3	0.3	0.7	0.3	0.3	0.7	0.3	3.0	2.3
CR_29_	Beautiful	2.0	2.7	3.0	3.0	2.3	3.0	0.3	0.7	1.7	1.7	2.0	1.7	0.3	0.3	2.0	3.0
CR_30_	Coordinating	2.3	0.0	1.3	3.0	1.0	3.0	1.0	2.3	1.3	0.3	1.0	0.7	0.3	0.3	2.7	2.3
CR_31_	Harmonious	2.3	0.0	1.0	3.0	1.7	3.0	1.3	2.3	1.0	0.3	1.7	0.3	0.0	0.3	2.0	2.3
CR_32_	Distinct	0.0	2.7	1.0	1.3	0.7	0.0	0.7	0.0	0.0	0.0	0.3	0.3	0.0	0.0	0.7	0.3
CR_33_	Sportive	0.0	0.3	0.3	0.0	1.3	0.0	1.3	3.0	2.0	0.0	0.7	1.7	0.7	0.3	0.0	1.3
CR_34_	Striking	2.0	0.0	2.0	0.3	0.7	0.0	1.7	0.3	0.3	1.0	0.7	0.0	3.0	1.3	0.7	0.7
CR_35_	Bionic	0.0	1.7	0.3	0.3	0.3	1.3	0.7	0.3	1.7	0.0	0.3	0.7	0.0	1.3	0.3	0.3
CR_36_	Avantgarde	3.0	2.0	1.3	1.7	1.0	1.0	1.7	0.3	0.7	0.3	0.3	3.0	1.7	1.7	0.3	1.3
CR_37_	Fickle	0.3	1.7	0.7	0.3	0.3	1.0	0.3	0.7	2.0	0.0	0.0	0.7	0.7	0.3	0.7	0.3
CR_38_	Dynamic	0.0	0.7	0.7	0.3	2.3	0.0	1.7	0.0	0.0	1.3	0.7	1.3	0.3	0.3	0.3	2.7
CR_39_	Technological	3.0	3.0	1.3	2.0	2.0	2.7	0.3	1.7	0.3	0.0	1.3	3.0	0.7	0.3	0.3	2.3
CR_40_	Likeable	0.0	0.0	0.3	0.3	0.7	0.0	1.0	0.7	2.3	2.7	0.3	0.7	1.3	0.0	0.3	2.3
CR_41_	Saponaceous	2.7	1.7	1.3	1.3	0.3	0.0	0.7	2.7	1.3	2.3	0.3	1.3	0.7	0.3	2.3	2.7
CR_42_	Gorgeous	0.7	0.3	2.3	1.7	0.0	0.3	0.3	0.3	1.0	0.0	0.0	0.0	0.0	0.3	0.0	0.7
CR_43_	Heavy	1.3	0.7	0.7	0.3	3.0	0.0	0.3	0.0	0.3	0.7	0.7	0.7	0.3	0.3	0.7	0.7
CR_44_	Abstract	0.0	1.7	0.3	0.0	0.3	0.3	2.7	0.7	0.0	0.0	0.7	0.3	1.7	1.0	0.0	0.7
CR_45_	Overbearing	1.3	0.7	1.3	0.7	3.0	0.0	0.3	1.3	0.7	0.7	0.3	0.7	0.3	0.3	0.3	0.3
CR_46_	Comic	0.0	0.3	0.7	0.3	0.0	0.7	0.7	0.0	1.7	1.7	0.3	0.0	0.0	0.3	0.3	2.7
CR_47_	Agile	0.3	1.0	0.0	1.3	0.3	0.0	0.3	3.0	0.0	0.0	0.3	0.3	0.7	0.0	0.3	0.3
CR_48_	Tensional	0.0	1.3	2.7	0.3	2.3	0.3	1.0	2.0	0.7	0.3	0.7	0.3	0.0	2.0	0.7	0.0

		CR_17_	CR_18_	CR_19_	CR_20_	CR_21_	CR_22_	CR_23_	CR_24_	CR_25_	CR_26_	CR_27_	CR_28_	CR_29_	CR_30_	CR_31_	CR_32_
		Bright	Hard	Glossy	Vivid	Organic	Fluent	Energetic	Powerful	Precise	Classy	Orderly	Decent	Beautiful	Coordinating	Harmonious	Distinct

CR_1_	Simple	0.3	1.7	2.0	0.7	3.0	1.0	0.7	0.0	1.3	0.3	3.0	3.0	1.0	0.0	0.3	0.0
CR_2_	Modern	0.3	0.7	2.3	2.3	3.0	1.3	0.3	0.3	2.3	0.0	0.0	1.7	1.3	0.0	0.0	2.0
CR_3_	Outstanding	0.3	0.7	0.0	2.3	0.0	0.3	0.0	0.3	1.7	3.0	0.0	1.3	2.7	1.0	1.3	3.0
CR_4_	Exquisite	0.0	0.0	0.3	0.3	0.7	0.7	0.0	0.7	2.0	0.0	1.0	0.7	1.0	2.0	1.7	0.3
CR_5_	Manly	0.3	3.0	0.7	0.3	0.7	0.3	0.3	3.0	0.3	0.0	0.3	0.3	0.3	0.3	0.7	0.0
CR_6_	Innovative	0.3	0.3	0.7	0.3	0.3	0.7	0.3	0.3	1.0	2.3	0.3	0.3	2.0	1.7	0.3	0.7
CR_7_	Free	0.7	0.3	0.3	0.3	3.0	3.0	1.0	0.0	0.0	0.3	1.3	0.0	0.3	0.0	1.7	0.3
CR_8_	Speedy	0.0	0.7	2.3	1.7	2.3	3.0	0.0	0.3	0.0	0.3	0.0	0.3	0.3	0.3	0.7	0.7
CR_9_	Lively	2.7	0.3	0.0	2.7	2.3	2.0	2.7	0.7	0.3	0.3	0.7	1.7	0.3	0.7	0.7	2.7
CR_10_	Plump	0.0	0.7	2.3	2.3	2.7	1.0	0.0	0.0	0.3	0.3	0.3	1.3	0.3	0.0	0.3	1.0
CR_11_	Elaborate	0.3	0.0	0.7	0.7	0.3	0.0	0.0	0.7	3.0	0.0	1.7	0.3	2.3	2.3	2.7	1.3
CR_12_	Futuristic	0.3	0.3	0.3	0.0	2.3	2.0	0.7	0.3	3.0	0.0	0.0	0.7	0.3	0.3	0.0	0.7
CR_13_	Special	0.0	0.3	0.3	1.3	0.0	0.7	0.0	0.7	0.3	0.3	0.3	0.7	0.7	0.7	0.3	1.0
CR_14_	Abrupt	1.3	0.0	0.0	1.3	0.7	0.3	0.3	0.0	0.0	0.3	0.3	0.7	0.3	0.3	0.7	2.3
CR_15_	Integral	0.3	0.7	1.7	0.0	1.3	0.0	0.7	0.3	0.3	1.0	2.7	2.7	2.7	2.7	1.7	0.3
CR_16_	Mellow	0.3	0.3	2.7	0.3	3.0	2.7	2.3	0.3	0.3	0.3	2.3	1.0	2.0	1.3	1.0	1.3
CR_17_	Bright	0.0	0.0	0.7	3.0	0.0	0.3	0.3	0.0	0.0	0.7	0.7	0.7	0.0	0.7	0.0	3.0
CR_18_	Hard	0.7	0.0	0.3	0.7	0.0	0.3	0.0	3.0	0.3	0.0	0.7	0.0	0.3	0.3	0.0	0.0
CR_19_	Glossy	1.3	0.3	0.0	0.7	2.0	2.3	0.0	0.0	0.3	0.0	1.0	1.3	0.0	1.3	0.3	0.3
CR_20_	Vivid	2.3	0.3	0.0	0.0	0.0	0.3	0.3	0.3	0.3	0.3	0.0	0.3	0.3	0.7	0.3	3.0
CR_21_	Organic	0.3	0.3	3.0	0.7	0.0	3.0	1.0	0.7	0.3	0.0	0.3	0.0	0.7	0.3	0.3	0.7
CR_22_	Fluent	0.3	0.0	3.0	0.3	3.0	0.0	0.0	0.3	0.7	1.3	2.3	0.7	2.0	1.3	2.3	0.0
CR_23_	Energetic	3.0	0.0	0.0	2.3	2.0	0.0	0.0	0.3	0.7	0.7	0.3	0.0	1.0	0.3	0.3	2.0
CR_24_	Powerful	0.7	2.0	0.3	0.0	0.0	0.0	0.7	0.0	0.0	0.7	0.3	0.0	0.7	0.7	0.3	0.7
CR_25_	Precise	0.3	0.3	0.7	0.0	0.0	1.7	0.3	0.0	0.0	0.0	0.0	0.0	0.3	0.3	0.0	0.0
CR_26_	Classy	0.3	0.0	2.0	0.3	0.7	0.3	0.3	0.3	0.0	0.0	1.3	0.0	3.0	2.0	2.3	0.7
CR_27_	Orderly	0.3	0.0	1.7	0.7	1.3	2.3	0.0	0.3	0.0	0.3	0.0	2.3	2.7	1.3	0.7	0.3
CR_28_	Decent	2.3	0.3	2.7	0.0	1.7	2.0	0.3	2.3	0.7	2.7	3.0	0.0	2.7	1.3	0.7	0.0
CR_29_	Beautiful	2.0	0.0	3.0	2.0	2.0	3.0	0.7	0.0	0.7	3.0	2.0	3.0	0.0	3.0	3.0	1.3
CR_30_	Coordinating	1.7	0.7	2.7	0.7	1.7	2.3	0.3	0.0	2.7	3.0	2.3	2.0	3.0	0.0	3.0	1.3
CR_31_	Harmonious	1.7	0.3	2.0	0.3	1.0	2.3	0.3	0.3	0.0	3.0	2.3	2.7	3.0	3.0	0.0	1.0
CR_32_	Distinct	3.0	0.3	0.7	3.0	0.0	0.7	0.3	0.7	0.3	0.3	0.3	1.3	0.3	0.3	0.3	0.0
CR_33_	Sportive	1.3	0.7	2.0	2.7	1.7	3.0	2.3	0.0	0.7	0.3	2.3	0.0	0.7	0.7	0.3	0.7
CR_34_	Striking	3.0	0.0	0.0	3.0	0.3	0.3	0.0	0.0	0.0	0.3	0.7	1.3	0.0	0.3	0.7	3.0
CR_35_	Bionic	0.3	0.0	0.3	0.3	1.7	0.0	1.3	0.3	0.3	0.3	0.7	0.3	0.0	0.3	0.7	0.7
CR_36_	Avantgarde	0.7	0.0	0.3	2.3	2.3	0.0	0.3	0.7	2.0	0.0	0.3	0.0	0.3	0.3	0.3	2.3
CR_37_	Fickle	0.0	0.3	0.0	0.3	0.0	0.3	0.3	0.7	0.7	0.3	0.0	0.3	0.7	0.7	0.0	0.3
CR_38_	Dynamic	3.0	0.7	2.7	2.3	2.7	2.3	2.0	0.3	0.3	0.7	2.0	0.3	0.3	1.3	0.3	2.7
CR_39_	Technological	2.0	2.3	1.3	0.7	1.3	1.3	0.3	2.0	3.0	2.3	2.0	2.3	0.3	2.3	0.3	0.0
CR_40_	Likeable	0.0	0.0	2.7	2.7	1.3	0.3	2.0	0.3	0.3	0.7	0.3	0.3	0.0	0.3	0.7	2.7
CR_41_	Saponaceous	1.7	0.0	3.0	0.0	3.0	0.0	0.0	0.3	0.0	1.3	1.3	1.3	2.3	0.3	0.0	1.3
CR_42_	Gorgeous	3.0	0.3	0.0	3.0	0.3	0.3	0.7	0.3	0.3	0.0	0.3	0.0	0.0	0.3	0.0	2.3
CR_43_	Heavy	0.3	3.0	0.3	0.7	0.7	0.0	0.7	3.0	0.0	0.3	0.3	1.0	0.3	0.7	0.0	0.3
CR_44_	Abstract	0.7	0.7	0.7	0.3	0.3	0.3	0.3	0.3	0.3	0.0	0.7	0.3	0.7	0.7	0.0	0.7
CR_45_	Overbearing	0.7	3.0	0.7	0.7	0.3	0.7	0.3	3.0	0.7	0.3	0.3	1.3	0.7	0.7	0.0	0.7
CR_46_	Comic	2.3	0.3	2.3	2.7	2.3	0.3	2.3	0.3	0.0	0.7	0.3	0.3	0.0	0.0	0.3	2.3
CR_47_	Agile	0.3	0.0	0.0	0.0	0.0	2.7	0.0	0.0	0.3	0.3	0.3	0.0	0.3	0.3	0.7	0.3
CR_48_	Tensional	1.0	2.7	0.3	0.0	2.0	0.3	1.7	0.3	0.3	0.7	0.3	0.7	0.0	0.7	0.0	0.0

		CR_33_	CR_34_	CR_35_	CR_36_	CR_37_	CR_38_	CR_39_	CR_40_	CR_41_	CR_42_	CR_43_	CR_44_	CR_45_	CR_46_	CR_47_	CR_48_
		Sportive	Striking	Bionic	Avantgarde	Fickle	Dynamic	Technological	Likeable	Saponaceous	Gorgeous	Heavy	Abstract	Overbearing	Comic	Agile	Tensional

CR_1_	Simple	1.0	1.7	0.0	3.0	0.3	1.0	3.0	0.3	2.3	0.3	0.3	0.0	0.3	0.0	2.3	0.0
CR_2_	Modern	2.7	0.0	2.3	3.0	0.7	2.0	3.0	0.3	2.0	0.0	0.3	3.0	0.3	0.3	2.7	0.7
CR_3_	Outstanding	0.3	2.7	3.0	3.0	1.0	2.3	2.7	1.3	0.7	3.0	0.3	1.3	3.0	0.0	0.3	3.0
CR_4_	Exquisite	0.3	0.3	0.7	0.7	0.7	1.0	3.0	0.3	0.7	0.3	0.0	0.7	0.0	0.3	0.7	0.3
CR_5_	Manly	0.7	0.3	0.0	0.3	0.7	0.7	0.7	0.7	0.3	0.7	3.0	0.0	3.0	0.0	0.0	3.0
CR_6_	Innovative	0.7	0.7	2.3	3.0	2.7	1.7	1.7	0.0	0.7	0.3	0.3	2.3	1.3	0.3	1.3	1.3
CR_7_	Free	2.0	0.7	2.3	2.3	0.0	2.3	1.7	0.7	2.7	0.0	0.3	3.0	0.3	2.3	2.7	2.3
CR_8_	Speedy	3.0	0.7	1.3	0.0	0.3	3.0	0.7	0.7	2.3	1.3	0.3	0.3	1.3	0.7	3.0	0.3
CR_9_	Lively	3.0	1.3	2.3	0.0	2.3	2.3	0.3	2.3	2.3	2.7	0.7	0.3	0.3	3.0	0.0	0.3
CR_10_	Plump	0.0	2.3	0.7	0.7	0.3	1.7	0.0	2.3	2.0	0.3	0.0	0.3	0.7	2.3	0.0	0.0
CR_11_	Elaborate	0.3	0.3	0.7	2.3	2.7	0.0	2.3	0.0	0.0	0.3	0.0	2.0	2.3	0.0	0.7	0.3
CR_12_	Futuristic	1.0	0.7	3.0	3.0	0.0	2.7	3.0	0.3	2.3	0.3	0.7	2.3	2.0	0.3	0.0	0.0
CR_13_	Special	0.3	1.7	0.0	0.0	0.0	0.3	0.0	0.3	0.0	1.3	0.7	0.0	1.3	1.0	0.0	0.0
CR_14_	Abrupt	0.3	1.7	0.0	0.0	0.3	0.0	0.3	0.3	0.7	1.7	0.0	0.0	1.0	0.3	0.7	0.0
CR_15_	Integral	0.7	0.3	0.3	0.3	0.3	0.3	1.0	0.3	2.7	0.3	1.0	0.3	0.7	0.7	2.7	0.7
CR_16_	Mellow	1.7	0.7	0.3	0.0	0.3	2.3	1.3	2.7	3.0	0.3	0.3	0.3	0.0	2.3	2.7	0.3
CR_17_	Bright	1.3	2.0	0.7	0.3	0.7	0.3	0.7	2.0	0.3	3.0	0.3	0.7	0.3	2.0	0.3	0.3
CR_18_	Hard	0.3	0.7	0.3	0.3	0.0	0.3	0.7	0.3	0.7	0.3	3.0	0.3	3.0	0.0	0.3	0.3
CR_19_	Glossy	0.0	0.3	0.7	0.3	0.3	2.3	0.3	2.7	3.0	0.3	0.3	0.0	0.3	1.7	0.0	0.7
CR_20_	Vivid	2.3	3.0	0.0	0.3	0.3	0.3	0.3	2.0	0.0	3.0	0.3	0.0	0.3	2.3	0.0	0.7
CR_21_	Organic	2.7	0.3	3.0	0.7	0.7	2.0	2.3	2.0	3.0	0.3	0.0	2.7	0.3	1.7	0.0	0.0
CR_22_	Fluent	3.0	0.3	2.3	0.0	0.7	2.7	0.3	0.0	0.0	0.3	0.3	0.7	0.0	1.3	2.0	0.0
CR_23_	Energetic	3.0	1.3	3.0	0.3	0.7	3.0	0.7	2.3	0.0	2.3	0.7	1.0	0.0	3.0	0.0	0.0
CR_24_	Powerful	0.7	0.3	1.0	0.7	0.0	0.3	0.3	0.0	0.0	0.7	3.0	0.0	3.0	0.7	0.0	2.3
CR_25_	Precise	0.0	0.3	0.3	2.3	0.3	0.3	3.0	0.7	0.7	0.0	0.3	0.3	0.3	0.3	0.7	0.3
CR_26_	Classy	0.3	0.7	0.3	0.0	0.3	0.0	3.0	0.3	0.7	0.3	0.3	0.0	0.3	0.7	0.0	0.3
CR_27_	Orderly	2.0	0.3	0.3	0.7	0.3	1.3	0.3	0.3	2.0	0.0	0.7	0.7	0.0	0.3	0.3	0.7
CR_28_	Decent	0.0	0.0	0.7	0.0	0.0	1.3	0.3	0.3	0.3	0.3	0.3	0.3	0.3	0.7	0.7	0.7
CR_29_	Beautiful	1.3	1.3	2.0	0.7	0.0	1.3	2.3	0.0	2.0	0.0	0.3	0.7	0.3	1.7	0.7	2.0
CR_30_	Coordinating	2.0	0.7	2.3	0.7	0.3	2.0	2.3	2.7	2.3	2.0	0.0	0.7	0.7	2.7	2.0	0.0
CR_31_	Harmonious	2.3	0.0	2.0	0.7	0.3	2.3	2.0	2.7	2.0	2.3	0.3	0.7	0.0	2.0	2.3	0.7
CR_32_	Distinct	1.7	3.0	0.0	0.3	0.7	0.3	0.0	2.7	0.3	3.0	0.7	0.0	0.0	3.0	0.0	0.0
CR_33_	Sportive	0.0	1.3	2.0	0.7	1.3	3.0	0.7	1.0	0.0	1.7	0.7	0.3	0.3	1.0	3.0	0.3
CR_34_	Striking	1.0	0.0	3.0	2.3	0.3	0.3	0.0	0.7	0.3	3.0	0.0	1.3	2.7	1.3	0.0	2.0
CR_35_	Bionic	2.3	0.0	0.0	1.0	0.0	1.3	0.7	0.3	0.3	0.7	0.0	1.7	0.7	0.0	0.0	0.3
CR_36_	Avantgarde	1.3	0.3	3.0	0.0	1.3	1.7	3.0	0.3	0.3	1.0	0.7	3.0	0.3	0.0	0.3	1.3
CR_37_	Fickle	0.3	0.3	2.3	0.0	0.0	0.7	0.3	0.3	0.0	0.3	0.3	0.3	0.3	0.0	0.3	0.3
CR_38_	Dynamic	3.0	1.3	2.7	1.7	2.0	0.0	0.0	1.3	2.0	2.3	0.7	0.7	0.3	2.7	3.0	0.7
CR_39_	Technological	2.7	0.3	2.3	2.0	1.3	2.3	0.0	0.3	0.3	0.3	0.3	0.3	1.0	0.3	0.3	0.0
CR_40_	Likeable	1.7	1.7	2.3	0.7	0.7	1.3	0.3	0.0	2.0	1.7	0.3	0.3	0.3	3.0	0.7	0.7
CR_41_	Saponaceous	0.0	0.3	1.3	0.0	0.3	2.3	1.3	2.0	0.0	0.7	0.7	0.7	0.3	2.7	0.0	0.0
CR_42_	Gorgeous	0.7	2.3	0.7	0.0	0.7	0.0	0.3	2.3	0.7	0.0	0.7	0.0	0.3	2.0	0.3	0.3
CR_43_	Heavy	0.7	0.3	0.3	0.0	0.3	0.3	0.3	0.3	1.0	0.3	0.0	0.3	0.0	0.0	0.0	0.0
CR_44_	Abstract	0.7	0.3	0.0	2.7	0.3	0.3	1.3	0.3	2.7	0.7	0.0	0.0	0.7	0.3	0.7	0.0
CR_45_	Overbearing	0.3	0.0	0.3	0.7	0.0	0.3	2.0	0.0	0.0	0.0	2.7	0.3	0.0	0.7	0.3	2.0
CR_46_	Comic	1.3	2.7	2.3	1.3	0.3	1.0	0.7	3.0	2.3	1.7	0.0	0.3	0.7	0.0	0.3	0.3
CR_47_	Agile	2.0	0.7	0.0	0.0	0.0	2.3	0.3	0.3	0.0	0.0	0.3	0.3	0.0	0.3	0.0	0.3
CR_48_	Tensional	2.3	1.7	0.3	0.0	0.3	2.3	1.3	0.3	1.7	1.0	0.0	0.7	2.3	0.7	1.0	0.0

**Table 3:** Computing results according to different number of clusters.

Number of clusters	Elapsed time	Converged generation	Max fitness
2	50.196708	33	47.2487
3	26.156143	135	60.2140
4	33.05028	128	69.3339
5	41.888425	149	78.2695
6	52.731830	142	82.2049
7	56.553871	168	85.3748
8	63.927597	109	81.6808
9	71.302712	151	88.3222
10	78.391867	166	87.6516
11	85.253257	166	87.6000
12	94.667694	182	88.1333

**Table 4:** The six clusters and respective adjectives.

Clusters	Kansei needs adjectives					
CL1	Modern	Outstanding	Exquisite	Innovative	Elaborate	Precise
	Classy	Coordinating	Harmonious	Technological	Orderly	

CL2	Speedy	Lively	Plump	Mellow	Glossy	Organic
	Fluent	Energetic	Sportive	Dynamic	Saponaceous	Agile

CL3	Futuristic	Special	Abrupt	Bionic	Distinct	Avantgarde
	Abstract	Tensional	Simple			

CL4	Manly	Hard	Powerful	Heavy	Overbearing	

CL5	Bright	Vivid	Striking	Likeable	Gorgeous	Comic
	Beautiful					

CL6	Free	Integral	Decent	Fickle		

## Additional Points

*Relevance to Industry.* This study presents a numerical design structure matrix approach to extract primary needs in product emotional design process. Specifically, combined with genetic algorithms, the research offers a convenient and efficient method for clustering Kansei needs and finding optimum clusters. It appears that the proposed method can be utilized to reduce the dimension complexity of Kansei needs.
